# Navigating Uncertainty: Early Career Academics and Practices of Appraisal Devices

**DOI:** 10.1007/s11024-020-09425-2

**Published:** 2020-12-14

**Authors:** Jonatan Nästesjö

**Affiliations:** Department of Educational Sciences, Lund University, Sölvegatan 16, 22100 Lund, Sweden

**Keywords:** Appraisal Devices, Uncertainty, Valuation, Identity, Socialization, Early Career Academics

## Abstract

There is a lack of objective evaluative standards for academic work. While this has been recognized in studies of how gatekeepers pass judgment on the works of others, little is known about how scholars deal with the uncertainty about how their work will be evaluated by gatekeepers. Building upon 35 interviews with early career academics in political science and history, this paper explores how junior scholars use appraisal devices to navigate this kind of uncertainty. Appraisal devices offer trusted and knowledgeable appraisals through which scholars are informed whether their work and they themselves are good enough to succeed in academia. Investigating how early career academics rely upon appraisals from assessors (i.e., ‘academic mentors’), the study adds to existing literature on uncertainty and worth in academic life by drawing attention to how scholars’ anticipatory practices are informed by trusting the judgment of others. The empirical analysis demonstrates that early career academics are confronted with multiple and conflicting appraisals that they must interpret and differentiate between. However, the institutional conditions for dealing with uncertainty about what counts in future evaluations, as well as which individuals generally come to function as assessors, differ between political science and history. This has an impact on both valuation practices and socialization structures. Focusing on what I call *practices of appraisal devices*, the paper provides a conceptual understanding of how scholars cope with uncertainties about their future. Furthermore, it expands existing theory by demonstrating how scholars’ self-concept and desired identities are key to the reflexive ways appraisal devices are used in the course of action.

## Introduction

Uncertainty is a main concern in academic life. Caused by a lack of knowledge about the outcome of actions, it is a problem that scholars must navigate when trying to reach ends. Previous research has acknowledged the significance in dealing with the inherent uncertainties to knowledge production (Knorr-Cetina 1999) and how the degree of task uncertainty varies across fields (Whitley 2000). Furthermore, there is a growing literature that, rather than focusing on the epistemic uncertainties in the research process, addresses how social uncertainties related to job security and careers are significant for the practices and identities of researchers (Gill 2014; Knights and Clarke 2014). Central to the latter perspective is to analyze how the conditions for doing and valuing research have changed under the impact of New Public Management. This includes new evaluative practices of research performance (de Rijcke et al. 2016), an increasingly competitive allocation of recourses (Roumbanis 2019), as well as changing temporalities inherent to the projectification of academic work and careers (Felt 2017; Ylijoki 2016).

Under these conditions, epistemic and social uncertainties become deeply intertwined and researchers are therefore compelled to address uncertainties related to the future in new ways (Fochler and Sigl 2018). This is especially evident for junior scholars aspiring to make an academic career. Yet to obtain a position, they are required to anticipate how their work will develop within limited time frames and which accountable outputs it will deliver (Fochler et al. 2016; Müller 2014; Sigl 2016). However, early career academics must also try to anticipate *what counts* as accountable outputs and its *relative worth* in specific institutional contexts. Indeed, recent studies within the field of science and evaluation studies have emphasized the importance of “resisting singular notions of ‘excellence’” and paying attention to how “academic research is becoming increasingly accountable to multiple – sometimes complementary, sometimes conflicting – evaluative infrastructures and regimes of worth” (Rushforth et al. 2019: 211). But how exactly does that work? Given that there is not singularity to (e)valuation in academia, how do junior scholars reduce uncertainty about what counts? And how do they use this kind of knowledge to evaluate their own performance and to guide their future work?

This paper investigates how early career academics in two disciplinary cultures within the social sciences and humanities cope with uncertainties about how to demonstrate one’s worth in accordance with what counts in future evaluations. Inspired by the works of Fürst (2018), I conceptualize this as a tension between *career aspiration* and *market uncertainty*. Career aspiration is about people wanting to advance and thus to secure a position within a specific field. Market uncertainty occurs when these people are uncertain about how their abilities and work will be evaluated by gatekeepers within the field. This tension is important to study, as it is present throughout academia. Because scholars produce unique and incommensurable ‘goods,’ there is a general lack of objective evaluative standards. While this has been recognized in studies of how academic gatekeepers pass judgment on the works of others (Hammarfelt and Rushforth 2017; Lamont 2009; Musselin 2010), little is known about how scholars deal with the uncertainty about how their work and they themselves will be evaluated by gatekeepers.

In the comparative case study presented below, I will demonstrate how early career academics in political science and history come to use different sources of information about quality, worth, and performance when dealing with the tension between career aspiration and market uncertainty. I propose to think about these sources of evaluative information as *appraisal devices* (Fürst 2018). Appraisal devices come from knowledgeable and trusted sources, where early career academics are informed about whether their work and they themselves are good enough to succeed in academia. In contrast to any form evaluation, appraisal devices are distinctive in that their usefulness depends on a *matching logic* between the evaluation that constitutes the appraisal and subsequent evaluations on the academic market.

While there are several sources of appraisal devices available to junior scholars, I am zooming in on how they use evaluations produced by what Fürst (2018) calls *assessors*. Assessors are trusted individuals who are part of a junior scholar’s network and who appraise his or her chances of success and failure. Because junior scholars trust the assessor’s ability to produce evaluations that correspond to the judgment of gatekeepers, assessors are able to act as stand-ins, evaluating scholars as if they were being evaluated on the academic market. Whereas the social function of an assessor may be described as an ‘academic mentor,’ the term does not refer to any employment positions and is not limited to any formal definitions of mentorship (cf. Eby et al. 2007). Instead, an assessor is any individual – a supervisor, a PI, a colleague, an editor, a reviewer, a friend – who the early career academics perceive as knowledgeable about how scholars are evaluated on the academic market and who are able to produce such evaluations.

By using appraisal devices, early career academics can temporarily reduce uncertainty, enabling them to make informed decisions about their research and careers. However, faced with multiple and conflicting appraisals, scholars must also interpret and differentiate between them. This may be understood as a form of boundary-work, where early career academics make distinctions between those whose judgment they do and do not trust. In this process, scholars’ self-concept and desired identities are key to the reflexive ways appraisal devices are used in the course of action.

Introducing the appraisal devices framework to the field of science and evaluation studies, this paper provides a conceptual understanding of how early career academics cope with uncertainties about their future. The ambition is to shed light on how junior scholars come to orient themselves within highly complex institutional environments by learning how to anticipate future evaluative criteria. Furthermore, analyzing the use of appraisal devices among those who are currently growing into academia opens up for understanding the entanglement of uncertainty reduction and academic socialization processes. In reducing uncertainty about how to demonstrate one’s worth in accordance with ‘what counts,’ appraisal devices both help to organize the world and observe oneself. As such, the appraisal devices framework offers a novel way of sensitizing how ways of coping with uncertainty not only yield certain ways of reasoning and preferences to act, but also ways of building one’s own academic identity.

## Early Career Academics: Uncertainty and Worth in Project-based Careers

Although there is no agreed upon definition for what constitutes an ‘early career academic,’ the label generally refers to academics in a phase of transition: from recently graduated PhDs to scholars with senior positions and stable employment (Haddow and Hammarfelt 2019). Considering the difficulty to delimit when scholars leave this career phase, the current study defines early career academics as a group of scholars who have received their PhD within the previous eight years and who are yet to obtain a permanent position. Working under fixed-term contracts, previous research has identified this group as particularly affected by both competition and changes in how academic work is evaluated and organized (Fochler et al. 2016). This includes studies of how junior scholars’ research practices and academic selves are increasingly shaped by neoliberal ideals (Archer 2008), performance indicators (Müller and Rijcke 2018), funding regimes (Roumbanis 2019), and policy measures such as mobility (Ackers 2008). Having to deal with a high level of job insecurity and pressing career norms, studies have also shed light on various social and political aspects of working as an early career academic, such as mental health problems (Signoret et al. 2019), emotion-work (Bloch 2002), and issues of gender discrimination (Murgia and Poggio 2018). Furthermore, literature focusing on academic mentoring has highlighted the importance of both social and human capital in academic careers. Providing junior scholars with training in specific skills, collaboration opportunities, and/or favorable networking opportunities, mentors have been found significant for novices reaching positions within academia (Bäker, Muschallik and Pull 2020).

While providing insights into the many-faceted reality of early career academics, these studies tend to deal with the problem of uncertainty rather implicitly. In fact, researchers across different fields have only recently begun to more systematically explore how shifts in the social organization of research produces and legitimizes a career system marked by uncertainty – and how this is experienced and handled by younger academics. Crucially, this includes scholars’ relationship to time, particularly the future (Felt 2017; Ylijoki 2010). According to Müller (2014), postdoctoral life scientists’ experiences of temporalities in the context of their work and career practices are increasingly shaped by both acceleration and individualization. Future uncertainties are addressed by aiming to improve units of outputs per units of time, fostering instrumental and tentative social relationships. Similarly, Sigl (2016) has shown that epistemic and social uncertainties become interlinked within the project as an organizational form. Focusing specifically on PhD students and postdocs in the life sciences, she argues that within project-based careers, the issue of future productivity comes to tacitly govern the research practices of the present, deeply affecting the rationales of younger scholars’ decision-making. Against this backdrop, Fochler and Sigl (2018) argue that the current organization of academic work produces what they call *anticipatory uncertainty*. Described as “the state of being uncertain of whether research processes will be productive in a specific timeframe and of how a specific institutional context defines performance” (Fochler and Sigl 2018: 350), anticipatory uncertainty captures the entanglement of epistemic uncertainties and social uncertainties. Comparing academic life sciences and biotechnology companies, their study demonstrates how diverse organizational logics result in highly different management of uncertainty in the research process.

Building on these prior works, this paper adds further dimensions to the study of uncertainty and worth in academic life. First, while previous studies have focused on the anticipatory practices of researchers, the appraisal devices framework provides a change in perspective, asking *how* these anticipatory practices actually are informed. Second, exploring how early career academics come to use and differentiate between appraisals when trying to establish themselves within their field provides an analytical opportunity for studying the entanglement of uncertainty reduction and socialization processes. Finally, studies of how scholars are affected by both uncertainty and the growth of research governance are heavily biased towards the life sciences. Focusing on political science and history, where career structures, working routines, and evaluative practices are shaped quite differently, will add new dimensions to our understanding of how early career academics experience and manage uncertainties when growing into academia.

## Theorizing Practices of Appraisal Devices

The concept of appraisal devices was coined by Fürst (2018) in his works on how aspiring writers deal with the uncertainty of not knowing whether their work is of the right quality to succeed in an artistic market. Being an extension of Karpik’s (2010) concept of *judgment devices*, it is part of the economics of singularities, that is, how the quality of unique and incommensurable ‘goods’ is determined. Examples of singularities are literary works, fine wines, and personalized professional services. Because these goods are characterized by quality uncertainty, judgment devices provide consumers with the credible knowledge needed to make and legitimate decisions. As has been argued elsewhere, academic evaluations provide a case in point since the quality of academic work, or an individual academic’s worth, is difficult to assess (Hammarfelt 2017). Similar to a film or a painting, a scientific article or an academic CV are defined by both incommensurability and quality uncertainty and may thus be understood as singularities (Karpik 2011). Accordingly, several studies have analyzed how judgment devices are used when making evaluative decisions in academic hiring processes (see e.g., Hammarfelt and Rushforth 2017; Hylmö 2018; Musselin 2010).

However, while judgment devices are about *consumption* of singularities, appraisal devices are about *production* of singularities. In the context of academic evaluation, this means that rather than focus on how gatekeepers (consumers) use devices such as rankings or the prestige of journals to legitimate judgment, appraisal devices focus on how scholars (producers) navigate the uncertainty of not knowing how they will be evaluated by gatekeepers on the academic market. As such, appraisal devices focus on scholars’ relationship to “markets in which they present and represent singularities” (Fürst 2018: 399), making it possible to theorize how they draw upon different sources of evaluative information as they try to succeed in careers conditioned by quality uncertainty.

Appraisal devices are distinctive in that they consist of evaluations that the producer of singularities can use in order to temporarily reduce uncertainty. In his work on aspiring writers, Fürst underlines that “appraisals only become useful appraisal devices when they come from sources that are both trusted and knowledgeable enough for the artist to assume that the appraisal corresponds to how the artist’s work will be evaluated on the artistic market” (Fürst 2018: 391). Hence, appraisal devices rely upon a *matching logic*. The *matching logic* highlights the anticipating function of appraisal devices. From the perspective of scholars, it is the ability of appraisal devices to turn *uncertainty* into *risk* that may facilitate action and decision-making. Indeed, both uncertainty and risk are shaped by the fact that the future is unknown. However, in contrast to situations of uncertainty, in which we cannot predict the possibility of a future outcome, situations of risk allow for such predictions to be made. This is because situations of risk are *calculable situations* (Knight 1921). Nevertheless, appraisal devices do not change the actual status of the situation, as uncertainty cannot be eliminated (Aspers 2018). Rather, appraisal devices function in so far as they let people act *as if* the future is more certain than it actually is (cf. Beckert 2016).

Consequently, for assessors’ appraisals to comply with the necessary *matching logic*, early career academics must *trust* their judgment. Based on personal trust, assessors are able to act as market intermediaries, producing evaluations that inform junior scholars whether their work and they themselves are good enough to succeed in academia. As demonstrated by Bessy and Chauvin (2013), intermediaries often have a double function in that they both provide actors with information and produce valuations on markets. This is especially true for assessors in academia who, just as literary critics, continuously switch roles between evaluating peers and being evaluated by peers. This social structure underlines the link between subjectivity and legitimacy in the production of artistic and academic judgment (Chong 2013). As a result, assessors are not neutral but part of producing and reproducing definitions of worth in academic fields.

Being primarily interested in mapping sources of appraisal devices, Fürst draws upon Kaprik’s theory about dimensions of judgment devices, distinguishing between different types of devices as well as diverse institutional arrangements that structure the dispersion of knowledge (Fürst 2018). While this provides him with a robust typology of appraisal devices, it does not let him pay sufficient attention to what I call *practices of appraisal devices* – that is, the processes by which actors engage with and interpret appraisal devices, including how they decide the criteria and means for using or disputing them in concrete situations. Indeed, the devices theory has recently been criticized for its reductive and functionalistic tendencies when applied in studies of academic evaluation (Kaltenbrunner and de Rijcke 2019). However, focusing on practices of appraisal devices, this paper tackles such criticism by emphasizing the reflexive work required by actors using them. Scholars’ responses to and use of appraisal devices are not mechanical, nor can they be reduced to mere calculation. Instead, they depend on how scholars interpret the appraisal and use it in the course of action. This may include factors such as disciplinary cultures and institutional conditions as well as personal experiences and the self-concept of scholars. Furthermore, while appraisal devices are part of producing and reproducing definitions of worth within academic fields, “there is no singularity to (e)valuation in academia” (Rushforth et al. 2019: 229). Consequently, the abilities of scholars and the quality of their work may be assessed according to different criteria, depending on the context of evaluation and the social position or taste of the evaluator. In practice, this means that early career academics often face the problems that arise from multiple appraisals competing with one another. In the absence of a clear standard of quality or success, they must decide whose judgment to trust. Hence, to navigate uncertainty, not only must scholars *interpret* the appraisals from assessors, they also need to *differentiate* between them.

Emphasizing the reflexive ways scholars use appraisal devices, the concept of *boundaries* is crucial for understanding how they come to trust and act upon the knowledge that assessors produce. As shown elsewhere, boundary-work are often symbolic, consisting of conceptual distinctions we make to “categorize objects, people, practices, and even time and space” (Lamont and Molnár 2002: 168). From this perspective, the *matching logic* is never given in advance but something that is negotiated in interactions between aspiring scholars and assessors. When established, this logic yields certain ways of reasoning and preferences to act. Hence, in studying practices of appraisal devices, this article provides an understanding of uncertainty reduction that is far from being a technical issue. Instead, it proposes to think about the use of appraisal devices as an interpretative and existential practice in which scholars come to make sense of the world they live in. This includes the many ways they shape their own practices and their academic selves by deciding whose judgment to trust.

## Material and Method

The two disciplines under study represent different epistemic cultures that share both similarities and differences. On the one hand, both history and political science are regarded as high status fields within the humanities and the social sciences, respectively, and as a consequence, the competition for funding as well as permanent positions is fierce. On the other hand, in the Swedish context, the two disciplines vary considerably in terms of common practices for publishing (e.g., publication language and publication genres) and collaboration (e.g., co-authorship and funding). These factors are known to influence how quality is conceptualized in intradisciplinary evaluations of academic careers (Hammarfelt 2017), including the ways researchers try to accumulate credibility (Hessels et al. 2019). While it is important not to underestimate the relative heterogeneity that characterize most disciplines in the social sciences and humanities today, it is equally important not to downplay existing differences. As argued by Paradeise and Thoenig (2013), general trends that are re-shaping the value regimes in academia might produce different local phenomena. From this perspective, political science and history in Sweden provide an interesting case. Whereas the praxis for doing and valuing research in political science have largely adapted to what is framed as ‘international standards,’ Swedish scholars in history have only recently begun to adapt to this trend. As a consequence, the current praxis for doing and valuing research in history has been described as *in flux* (Salö 2017).

With this in mind, the sampling strategy was designed to fulfill two purposes: to allow for a comparative analysis between the two disciplines and to bring as much variation as possible into the material. I therefore included junior scholars working at different universities and in different early career positions, with diverse experiences of research, teaching, and administration as well as experiences of publishing, mobility, and collaborative work. In total, the study includes two political science departments and three history departments located at four research-intensive universities in Sweden. To build a sampling frame, a list of early career academics in political science and history currently working at the selected departments and who had received their PhD between 2011 and 2019 was constructed. By consulting online profiles and CVs, I gathered information about the scholars’ age, gender, current and previous positions, track record for funding, list of publications, as well as scholarly awards. Based on a descriptive analysis of the gathered information, early career academics who worked under fixed-term contracts with the aim of bringing as much variety as possible into the material were sampled.

This procedure resulted in 35 interviews, conducted between February and June in 2019. Out of these interviews, 30 were conducted face to face and five were conducted through video link. A close to equal number of historians (9 male and 8 female) and political scientists (11 male and 7 female) were interviewed. A majority of the interviewed scholars worked at least part time in research positions, meaning that they had either received external funding or were employed in someone else’s research project. The interviews were conducted in Swedish or English and lasted between 90 and 140 minutes. All of the interviews were recorded and fully transcribed. Informed consent was obtained before each interview, which assured the respondent of voluntary participation and anonymity. Consequently, details that might make a respondent recognizable, such as specific area of research, name of university, or nationality, have been left out of the empirical sections.

The overall aim of the interviews was to gather data on how norms, values, and structures of making an academic career affect the practices and identities of junior scholars in political science and history. Structured as a reflexive biographical interview (cf. Sigl 2016), it had a specific focus on retrospective and prospective reflections about their own motivation to do academic work and how their aspiration to succeed in academia influenced their planning and decision-making. Furthermore, I continuously asked the respondents to draw boundaries between what they considered to characterize successful and unsuccessful academics – in terms of epistemic practices, career strategies, and other status traits. Inviting scholars to draw such boundaries from an internal perspective (what traits are the most valued by themselves) as well as an external perspective (what traits are the most valued by their academic environment and their discipline) allowed insights into how scholars’ decision-making is negotiated, not only in relation to perceived career expectations and conceptions of worth but also their social identity – that is, their self-concept and how others define them.

The objective for using retrospective and prospective interview questions was not to reveal how the respondents’ lives actually were or will be. Rather, the aim was to use temporal and situational perspectives as interview interventions for studying how early career academics conceptualize their room for maneuvering when growing into academia and what recourses they draw upon when organizing their research and careers. In this regard, a central part of the interviewees’ narratives concerned how to correctly demonstrate one’s worth in accordance with what counts in future evaluations. After going through a first cycle of inductive coding, which involved how scholars talked about their aspiration to succeed in academia and how they experienced and dealt with career uncertainties, I therefore moved on with a second cycle of coding using the central concepts of this article – appraisal devices, market uncertainty, career aspiration, and symbolic boundaries – to analyze the data. Several sources of appraisal devices were identified. However, in this article I zoom in on the one that was most frequently used, namely, assessors.

## Findings

The findings section is structured around the ways in which early career academics in political science and history deal with the tension between career aspiration and market uncertainty by using appraisal devices in the form of assessors. It begins with a description of how and under what conditions assessors come to function as appraisal devices, including how they are part of structuring the socialization processes differently within the two disciplines. Thereafter, the focus turns to how scholars interpret and differentiate between appraisals. The analysis demonstrates that identity, morality, and imagined futures are key to the practices of appraisal devices. The findings from the two disciplines are presented separately.

## Political Science

### Senior Assessors and Vertical Socialization Processes

The experience of uncertainty was a central topic in all of the conducted interviews with early career academics. Accounts of uncertainty experiences most often referred to the structure of, and the conditions for, qualification. Aspiring to succeed with an academic career, they all shared a feeling that there is a need for them to organize their work in relation to future evaluations. When talking about this aspect of being an aspiring scholar, they described different ways in which they actively searched for information about quality definitions and performance criteria; information that may, at least temporarily, reduce uncertainty and enable informed decisions about their research and careers. In this regard, what is characteristic for early career academics in political science are the importance and the temporal durability of appraisals given to them by senior scholars. Consider how this postdoc described his relationship to senior scholars during his time in academia:

I have worked with several professors: as a research assistant, as a PhD, and now as a postdoc. […] One professor in particular, who I met as a PhD student, he was not my supervisor but more like a mentor. […] He was the one who gave me feedback on my first article manuscripts, telling me what journals I should aim for. And he explained to me that I should aim for really high-ranked journals, both because that is what really counts when others evaluate your performance and because he thought that I had what it takes to publish there. (PS9m)

The most commonly used appraisal device by early career academics in political science is a senior scholar whose social function may be described as an ‘academic mentor.’ From the above quotation, we learn that the appraisals from a senior assessor can be used both to understand what counts on the academic market and for assessing the aspiring scholar’s own abilities and the quality of his work. In line with this, several respondents concluded that “without a senior scholar by your side, you’re lost” (PS8f). Because of their professional status, senior assessors are regarded as knowledgeable about how political scientists are evaluated on the academic market. Relying on a *matching logic*, they thus provide personalized knowledge about scholars’ chances of success and failure. However, this relationship is not only supported by formal status hierarchies, but also by the institutional conditions for pursuing an academic career in political science. Several of the interviewees described how they had been recruited by a senior researcher to work as their assistant before being encouraged to apply for a PhD position. This was generally interpreted as a sign of recognition that emotionally charged the aspiring scholar to sustain his or her aspiration of success. Furthermore, it provided them with a source of information about how to qualify themselves within the field:

Working together with my supervisor, first as an assistant and then as a PhD student, I learned from the beginning that there is some kind of ‘right way’ to do it. Directly, I was instructed to pave the way for the postdoc period, which meant to write in English and not in Swedish, to focus on research, not ending up teaching too much. Ehm… To write my dissertation so that it could easily be re-written and published by an international academic publishing house, and to publish one or two articles during my PhD. Yeah, then I would’ve ticked most of the right boxes. (PS15m)

Crucially, this also holds true for the time after completing the doctoral studies. In Sweden, as elsewhere (Franssen and de Rijcke 2019), it is a common practice for postdocs in social science disciplines to work in temporary projects run by a senior researcher. This gives early career academics immediate access to an assessor who may be used as a personal device for self-evaluation and future orientation. A concrete example is this quote from a junior scholar working in a project that runs over four years:

I had planned for four articles, one article per year in really, really good journals. Because I also have teaching duties, I thought this would be a good standard. But my PI said it was not good enough, that we should work on more stuff. And you know, he is really successful, he knows what it takes to make it at this level, so now I have changed my initial plan. (PS6f)

For this scholar, consulting the PI of the project in which she works reduced uncertainty about performance criteria at the postdoctoral level. Based on reputation and a successful track record, she trusted the assessor’s ability to anticipate future evaluative criteria and therefore used this knowledge to re-plan her research for the coming four years, aiming to further increase her productivity in terms of publications. While previous studies have focused on how working in temporary projects increases anxiety and career uncertainty (Franssen and de Rijcke 2019; Sigl 2016), the appraisal device perspective demonstrates that it may also be used as a resource for dealing with this kind of uncertainty. This was confirmed in the accounts of early career academics working in individual projects and who felt disorientated because of the lack of senior guidance:

Comparing to when I was a PhD student, I have no one to ask. I do not have a mentor or boss or anything. […] I can do this project the way I want to, but I am also on my own. And that is hard. I am constantly questioning myself and the work that I do, like, is this the right way to do it? Is it good enough? (PS4m)

While working in individual postdoc-projects provides aspiring scholars with independence and freedom, the feeling of being ‘on your own’ may also increase the experience of uncertainty. According to the above quotation, the absence of a senior assessor stands in stark contrast to the resources this scholar had for planning, doing, and valuing his work at the PhD level. Hence, the access to certain sources of appraisal devices are not the same across the social settings in which early career academics grow into academia. Furthermore, what sources of appraisal devices the individual scholar has access to may change over time.

In producing appraisals recognized as appraisal devices, senior assessors continuously act as intermediaries for gatekeepers on the academic market. Being regarded as both knowledgeable and powerful agents, early career academics in political science actively seek their appraisals for both recognition and future orientation. Consequently, the socialization processes in political science may to a large extent be understood as *vertically structured*, echoing a Bourdieusian conception of academic socialization in which dominating agents brings stability to prevalent power structures and interests by imposing dominating evaluative criteria (Bourdieu 1988). According to Musselin and Beckert (2013: 20), “the ability to impose criteria for quality evaluation is important because these become increasingly entrenched by their use.” Hence, in aspiring scholars’ use of appraisal devices, the criteria imposed by senior academics becomes accepted measures of quality and success within the field of political science.

### Practices of Appraisal Devices: Symbolic Communities and Notions of Trust

In typological terms, assessors belong to what Fürst (2018) calls *the network-market plus professional authority regime.* Used as a personal and substantial device, assessors are able to act as stand-ins to evaluate scholars’ work as if it were being evaluated by gatekeepers on the academic market. However, shifting focus from the typology to the practices of appraisal devices, it is evident that not every senior scholar in political science may function as an assessor. Because early career academics are faced with multiple appraisals, they need to draw boundaries between different sources of evaluative information when deciding which appraisal device(s) they will actually use. Regarding assessors, these boundaries are often drawn on the basis of *identification* with *symbolic communities*. This provokes an interesting divide regarding the establishment of trust. For some respondents, the practices of appraisal devices mainly revolve around their active search for appraisals from academics that they regard as exceptionally successful and competitive. Consider this quote from an experienced postdoc discussing how he differentiates between assessors:

For me, it pretty soon became clear that, if I want to be the best, I have to learn from the best. And this may sound cocky, but I do not want to become just an average political scientist who feels sorry for himself because others have had greater success. I want to become a top scholar. Given that, I have always sought the response and recognition from those who are regarded as top scholars. […] We have a couple of professors at the department who publish in absolute top journals, who work with scholars at the best American universities, who receive the most prestigious grants. You know, they kind of belong to an academic elite. For me, these are the ones that know how to make it; these are the ones whose judgment I trust. (PS14m)

For respondents to whom this way of reasoning is characteristic, being recognized as belonging to an academic elite is comprehended as a sign of trustworthiness, strengthening the *matching logic* between assessors’ appraisals and future evaluative criteria. Confronted with the problems caused by the need to decide between multiple appraisals, these scholars make distinctions between assessors depending on their reputation, understood primarily in terms of formal scholarly merits. In this process, the selection of a trustworthy assessor involves both the self-concept of the aspiring scholar and the symbolic community that the assessor represents.

This is also true for another group of respondents. Nonetheless, the boundaries produced when they decide whose judgment to trust are different. For this group, belonging to an academic elite cannot in itself result in a *matching logic*. Instead, these scholars tend to make critical remarks on how those belonging to academic elites are “over-achievers” (PS2f), someone that “just works 24/7 and do not have a real life” (PS8f), or individuals characterized by being “extremely unconscionable when it comes to making career advancements” (PS1m). In line with this, several respondents differentiate between assessors by drawing moral boundaries:

For me, trust comes from being a role model. And I mean this both in terms of being a productive and skillful researcher, but also a good person. Someone that is ambitious, but also cares about others… Not someone that sees everything as a competition. […] I actually do not care that much about the superstars we have here at the department, because the way that they seem to live their lives… That is not how I want to live my life as an academic. (PS2f)

According to this scholar, being perceived as trustworthy does not only rely on the formal scholarly merits of the assessor. The assessor’s ability to signal virtues such as work-life balance and empathy are also key. Because these are virtues that the aspiring scholar wants to identify with when imagining a future life in research, they are used to differentiate between assessors. Reputation is thus a central mechanism for the selection of assessors among early career academics in political science. However, what status traits translate into recognized appraisal devices differ considerably.

In analyzing practices of appraisal devices, we must take into account the reflexive ways scholars use them. Trying to orient themselves in situations where there is a lack of clear evaluative standards, early career academics are people who aspire to become something. This means that acts of uncertainty reduction should not be understood as a one-dimensional way of reducing the complexity of choice. Rather, it is an interpretative process in which appraisal devices provide aspiring scholars with justifications to decide between competing normative possibilities. Appraisal devices are thus not neutral but active forces; a form of valuation that establishes the worth of academics and their work. However, “for valuation, not only do people’s views matter; who these people are matters, too […] conceptualized in terms of identity, each has more or less status” (Aspers 2018: 140). This is to say that the practices of appraisal devices are influenced by how high-status traits are defined within specific contexts or groups, underlining the importance of the more or less stable status hierarchies and institutional role structures that order academic environments. The establishment of trust is thus not only a question about knowledge but also about identity, and the practices of appraisal devices involve the many ways early career academics interpret and engage with, as well as morally and culturally accept the authority behind appraisals.

## History

### Younger Assessors and Horizontal Socialization Processes

When navigating uncertainty by learning how to anticipate future evaluative criteria, early career academics in history often described their situation as characterized by “big changes” in terms of “how to compete successfully on today’s academic labor market” (H9f). Picturing their own conditions for doing and valuing academic work as radically different than the ones under which an older generation of historians were socialized, this had a substantial impact on whose judgment they generally come to trust:

They [senior scholars] may of course be good historians and I can still get good comments from them at seminars. But it is obvious that they have no idea what it means to be a junior scholar in academia today. […] In order to evaluate my work and to make decisions about the future, I talk to younger academics, either postdocs or someone that just got an employment. I never, and I mean never, consult senior scholars about these kinds of things. (H1m)

In the field of history, the most common assessor is not a senior academic but a more or less junior one. With reference to a ‘generation gap,’ the aspiring scholar above has learnt that there is a difference between evaluating research in general and evaluating it in relation to what counts on the academic market. While the former may include helpful comments about archival work, language, or general knowledge about the historical period under study, the latter are appraisals that aspiring scholars can use in order to assess and organize their own work in relation to future evaluations. This distinction was a recurrent theme in the interviews with early career academics in history. In the following, a newly minted PhD describes how she came to learn about this distinction in the course of the daily interactions at the department. After receiving positive comments at a seminar where she had presented a manuscript to be sent to an international journal, she met up with a more experienced postdoc who functioned as her mentor. He evaluated the manuscript differently:

He said that the text was good and interesting but that it was too book-like. There were too many arguments in the text and the research questions were way too focused on the Swedish context, so it lacked in relevance for an international audience. […] He said that it would most certainly be rejected, so I decided to re-write it quite substantially. (H10f)

For this scholar, it was the younger assessor who introduced the distinction between what is ‘good’ and ‘interesting’ in general terms from what is determined as quality from the perspective of the academic publishing market. Because the manuscript did not comply with the quality standards of the latter, he determined it not good enough and the aspiring scholar therefore continued to work on it. This means that an effective appraisal device cannot be based on just any other perspective. In comparison to studies demonstrating the variety of support academic mentors may provide junior scholars with (Bäker et al. 2020), the empirical analysis shows that early career academics, just like aspiring fictional writers (Fürst 2018), differentiate between possible mentors depending on whether or not they are able to produce evaluations according to the necessary *matching logic.* It is with reference to this logic that early career academics in history generally trust the appraisals from younger assessors who they comprehend as “knowledgeable about how things work in academia today” (H8f).

Early career academics in history described their postdoctoral position as being particularly precarious since it was not experienced as a natural continuation from their previous position as a PhD student. Rather, the criteria for how to qualify oneself as a successful scholar differ substantially between the two career phases. This particular kind of uncertainty experiences motivates the generation gap narrative:

From the day I received my PhD, the judgment of my supervisors basically became irrelevant. I still have a good relationship with them, but at this stage, they do not mean anything to my career or my research. This is simply because they do not have the competence… They do not publish peer-reviewed articles, they do not publish in English, or they do not publish at all… They do not compete for funding, so how could they possibly say anything about the quality of my work or how I should do things in order to succeed? For that, I have other, more or less junior researchers, who have both the experience and competence of doing these things (H12m)

In this quote, the aspiring scholar describes his transition from PhD to postdoc as characterized by the loss of trust in the appraisals given to him by his former supervisors. Regardless of the formal status position held by these senior academics, he did not comprehend them as trustable and knowledgeable enough to provide him with knowledge about his chances of success and failure at the postdoctoral level. Instead, to assess his own abilities and the quality of his work, he came to use appraisal devices in the form of younger assessors whose evaluations conform to the *matching logic*. Hence, who are able to produce appraisals recognized as appraisal devices may change over time. For early career academics in history, this happens because new gatekeepers, such as reviewers for international academic journals or funding panels, enter the field at the postdoctoral level, affecting the evaluation landscape in which they must navigate. In other words, when the conditions for gaining recognition change in connection with making career advancements, the conditions for using appraisal devices change with it.

According to Salö (2017), the current changes in publication and evaluation practices within the field of history in Sweden open up for junior scholars to invest differently than their senior peers. This is supported by the findings in this study. From the perspective of appraisal devices, it is evident that senior academics successively function less as market intermediaries. Instead, as junior scholars progress in their career, they more frequently come to rely upon the judgment of younger assessors. In this process, new criteria for evaluation are imposed and reproduced. Hence, as junior scholars grow into academia, the socialization structures in the field of history transform: from the vertical socialization processes that dominate the PhD education to the increasingly *horizontally structured* socialization processes that characterize the realities at the postdoctoral level.

### Practices of Appraisal Devices: Risk Management and Balancing Identity Positions

The conditions for dealing with uncertainties regarding quality definitions and performance criteria within the history field are profoundly shaped by the problems caused by the multiplication of appraisals competing with one another. Nearly all the interviewed scholars in history shared a preoccupation with, and somewhat of an anxiety about, the lack of consensus regarding how one should behave in order to accomplish a successful career within the field. This complexity was further accentuated by the fact that, although senior academics seldom function as assessors, they still act as gatekeepers on parts of the academic market. Consequently, ‘what counts’ was perceived as more person-biased, and thus harder to anticipate, in history than in political science.

What does this mean for the practices of appraisal devices in history? Faced with multiple and competing appraisals, one way of deciding what appraisal device to actually use, and how to use it, was to interpret it as a form of risk management:

I have a feeling that it is different things that counts depending on the situation. When applying for funding, it is international journal publications and previous grants. But when applying for a position, the second book can still be very important. So it is hard to know how to compete successfully in history, I would say. […] Careers can still look quite different within our field. And because of that, I want to have someone that can evaluate my CV from different perspectives. (H6m)

This quotation underlines the fact that early career academics must be active in their search for information about evaluative criteria. For the aspiring scholar above, this means that the establishment of trust is closely connected to identifying assessors who can act as intermediaries for both well-established and emerging regimes of worth. As such, the practices of appraisal devices are centered on assisting him in his effort to satisfy coexisting, and more or less conflicting, definitions of worth. In the field of history, this was a common strategy for adapting to what were experienced as dominant extra-disciplinary trends of academic evaluation and at the same time act in accordance with more traditional evaluation practices within the discipline. However, in the course of the interview, the above respondent continues:

I do trust this new generation of historians that are starting to get positions in the field. I think it is just a matter of time, really. We see this transformation in academia, in the humanities, and in history. We start to evaluate academic work differently. And as a junior scholar, you need to be aware of that and act accordingly. (H6m)

In using appraisal devices as a form of risk management, the *matching logic* involves a prediction of how the disciplinary field will develop in the near future. Evidently, this may put more focus on the emerging, rather than the traditional, evaluative criteria within the discipline. While it is important to note that this varied between the respondents in history, some scholars engaged in risk management by paying attention to the evaluative practices within other fields that they regarded as relevant for the development of their own:

When talking to other junior scholars in fields such as political science, sociology… fields that have some kind of kinship with history. These disciplines have already gone through this process of internationalization and it is quite clear what pays off in terms of making a career. So in order to understand where we are heading as a discipline, I pay attention to the state of affairs in other disciplines and how they evaluate academic work. (H1m)

According to Mennicken and Sjögren (2015: 4), much valuation “relies on the projections, estimates, and more or less systematically organized guesswork, which are invested in aspiration and hope.” As both of the quoted scholars above make clear, this is also true for the use of appraisal devices. Providing early career academics with information about what counts in future evaluations, practices of appraisal devices are always anticipatory practices, involving a projection of future value. When experiencing your own disciplinary field as ‘changing,’ these projections can be informed by comparing one’s abilities and work with the evaluation of junior scholars in other disciplines. As a compliment to existing appraisal devices, the ‘fictionality’ of such comparisons may be used in the task to reduce uncertainty when orientating one’s action towards the future.

Differentiating between assessors is not only a balancing act between competing definitions of worth, but also in terms of identity positions. As evident in political science, the self-concept of aspiring scholars and the symbolic community that assessors represent are key to the practices of appraisal devices. For most historians, this translates into questions about the relationship between strategic awareness and adaptability, on the one side, and authenticity and disciplinary identity, on the other. In the following, a postdoc explains why she trusts the ability of an assessor to evaluate her work from the right perspective:

H8f: I trust her judgment. You know, she has already written three books, she has published in international journals, she has won prestigious awards, she has received funding, and all that… So yeah, by every measure of the book, she is truly a successful scholar… But she is also someone that is honest and sincere, she is not just a careerist.

JN: Ok, is it important that she is not a careerist?

H8f: It is very important… I think you can learn how to do the right things, but you also need to learn how to do it the right way. A way that fits you and the type of academic you wish to be. But also an academic that others will respect, and in history, we generally do not respect careerists [small laughter].

In the account of this aspiring scholar, the establishment of trust relies both on formal status traits, such as scholarly awards and track record for funding, and on the perceived moral character of the assessor. This means that assessors must not only be able to demonstrate the ability to predict how scholars will be evaluated by gatekeepers, they must also uphold certain academic ideals, a way of *being* an academic that junior scholars want to identify with when trying to establish themselves within their field. Hence, to master the *matching logic*, assessors must be considered as credible in the eyes of the aspiring scholar and in the eyes of a more abstract, idealized disciplinary community by which the aspiring scholar wants to be recognized. This way of reasoning was particularly dominant when differentiating between assessors in terms of a distinction between academics being ‘fake’ or ‘true’:

We got these two very successful younger scholars at the department. They both publish a lot and their track record for receiving funding is impressive. So in a sense, they both know how to compete successfully. But one of them, he is… he is kind of fake. It is like… he knows what looks good on a CV, he has got all these publications, but he does not know how to produce really good research… When wanting comments on my work or discussing how to make it in academia, I never go to him for answers. (H14m)

Previous research has identified the concept of authenticity as a significant characteristic of what it means to work as an academic (Cannizzo 2018). From the perspective of appraisal devices, it plays an important part letting early career academics draw distinctions between individuals who may and may not function as assessors. In the quotation above, knowledge about how to compete successfully is not enough for the junior scholar to trust someone else’s judgment. Instead, an assessor must be able to correctly embody certain high-status traits that signal a shared and valued academic identity. Hence, it is by drawing symbolic boundaries that aspiring scholars come to recognize and act upon the *matching logic* between assessors’ appraisals and future evaluations.

## Discussion

This paper has investigated how early career academics in two disciplinary cultures use appraisal devices in the form of assessors to deal with the tension between their aspiration to succeed with an academic career and the uncertainty of not knowing how they will be evaluated by gatekeepers on the academic market. As such, the study has provided an analysis of how junior scholars come to orient themselves within highly complex institutional environments by learning how to anticipate future evaluative criteria. Focusing on how scholars in political science and history use assessors as a device for self-evaluation and future orientation, I have argued that the use of appraisal devices should not be understood as a one-dimensional way of reducing the complexity of choice. Instead, the *practices of appraisal devices* involve the many ways early career academics actively interpret and engage with, as well as morally and culturally accept the authority behind appraisals. For the assessor’s appraisals to correspond to the necessary *matching logic*, both knowledge and identity come into play in the establishment of trust. This is because appraisal devices do not only provide an orientation to navigate uncertainty. They also help scholars to observe oneself and to build one’s own academic identity.

The empirical analysis has shown that the conditions for using appraisal devices, as well as which individuals generally come to function as assessors, differ between the two disciplinary cultures under study. In political science, the most common assessor is a senior academic. With reference to formal status hierarchies and the institutionalized role structures that order the academic environments, aspiring scholars in political science trust the ability of senior assessors to anticipate future evaluative criteria. In actively seeking appraisals for both recognition and to inform their own research and career practices, the evaluative criteria imposed by senior academics become accepted measures of quality and success. The socialization processes in political science may therefore be characterized as predominantly *vertically structured*. As a prime example, the empirical analysis demonstrated that senior assessors are able to act as market intermediaries across different career levels, thus playing a vital role in the social reproduction of judgment in the field. Due to this temporal consistency, early career academics in political science have a quite coherent notion of how to qualify themselves, that is, how to correctly demonstrate one’s worth in accordance with ‘what counts.’

By contrast, the conditions for using appraisal devices within the field of history were shaped by an experience of profound discontinuation between how to demonstrate one’s worth in accordance with ‘what counts’ on the PhD and postdoctoral level, respectively. With reference to a growing generation gap in terms of both epistemic and evaluative practices, senior academics gradually function less as market intermediaries. As junior scholars in history progress in their career, they instead come to use younger academics as the primary form of assessors. In contrast to the vertically structured socialization processes that dominate the PhD education, the socialization processes at the postdoctoral level may thus be characterized as increasingly *horizontally structured*. However, because of the tension between using appraisals from younger assessors as appraisal devices and the institutional role structures ordering their academic environments, junior scholars in history are accountable to multiple, and sometimes conflicting, definitions of worth.

While this observation resembles what Stark (2009) labels ‘heterarchy,’ providing aspiring scholars in history with the possibility to switch between different definitions of worth when pursuing an academic career, it also makes future evaluative criteria harder to anticipate. According to Brandtner (2017: 203), “evaluations make essential paths and values visible by providing a cognitive map for social action.” Nevertheless, when scholars experience contradictory evaluative practices within their own field, evaluations do not make for a guiding cognitive map. In these situations, appraisal devices may be used as a form of risk management. In the history field, practices of appraisal devices often revolve around scholars’ effort to satisfy coexisting, and more or less conflicting, regimes of worth. This includes a projection of the future state of the discipline, where imagined futures form the background for valuation. As such, to understand scholars’ relationship with academic markets, we must pay attention to the temporal dimensions of using appraisal devices, in which the orientation towards the future is crucial.

The analysis resonates in several ways with recent studies of the anticipatory practices of researchers and the impact of new monitoring practices and the projectification of academic work (Felt 2017; Müller 2014; Ylijoki 2010). However, this paper provides a change in perspective, asking *how* such anticipatory practices actually are informed – that is, how early career academics come to learn about ‘what counts’ by deciding whose judgment to trust. From this perspective, the task is not only to analyze what practices academics put in place in order to manage uncertainty, for example, in terms of portfolio strategies (Rushforth et al. 2019) or organizational logics (Fochler and Sigl 2018), but how these anticipatory practices rely upon sources of evaluative information which scholars must differentiate between and act upon. This, in turn, opens up for a more complex understanding of how scholars deal with uncertainty and the co-existence of multiple definitions of worth.

Analyzing how early career academics interpret and draw boundaries between appraisals lets us pay attention to how they use appraisal devices both to navigate uncertainty and to build one’s own academic identity. As the empirical analysis demonstrates, the *matching logic* is not self-evident. Instead, it depends on a variety of factors, including how disciplinary ideals, high-status traits, desired identities, and perceptions of morality are negotiated. To capture this dynamic, I have focused on what I call *practices of appraisal devices* which provides an interpretive and existential understanding of how actors deal with uncertainty about their future. In doing so, this article extends previous work on ‘devices’ by showing how these are always *justificatory devices*, providing scholars with categorizations and legitimations of how to correctly *do* academic work and how to correctly *be* an academic. As such, appraisal devices have a double function in helping scholars to organize the world and to observe oneself. By acting upon the knowledge appraisal devices produce, scholars do not simply reduce uncertainty, they also signal their belonging to groups and shape their own identity through these group-memberships.

This development of the devices framework provokes new avenues of investigations. First, if scholars’ self-concept and the symbolic boundaries they draw are vital to the practices of appraisal devices – how are such conceptual distinctions produced and acted upon in diverse epistemic cultures? And what does this mean for what kind of knowledge that is likely to be produced? Studies situating uncertainty reduction more explicitly in epistemic terms would help to further crystallize the usefulness of the concept for theorizing the relationship between knowledge production and identity work. Second, because scholars’ responses to and use of appraisal devices depend on how they actively interpret the appraisals and use them in the course of action, future research should pay attention to how such interpretative practices are influenced by the cultural and social resources actors do, or do not, have access to. This would open up for an understanding of how a career system marked by uncertainty relates to institutionalized social differences (e.g., class and gender). Third, appraisal devices are not neutral but part of producing and reproducing definitions of worth in academic fields. In the case of assessors, this is further accentuated by the fact that they might continuously switch roles between acting as a mentor, a gatekeeper, an employer, and a scholar. The politics of appraisal devices, including the influence of social relations in academic judgment, needs further explorations. This may include questions about mentoring and networks, academic bias and nepotism, as well as social stratification in academia. Finally, on a more general level, practices of appraisal devices provide a conceptual framework for studying how actors orient themselves and develop their identity in fields where (i) concepts such as quality and worth are hard to define, and (ii) where gatekeepers play a significant role in determining the future of careers. This does not only include academia but also artistic and cultural markets as well as sports industries. Comparing how uncertainty of ‘what counts’ are dealt with in such diverse settings would put more focus on knowledge accumulation, making it possible to refine and develop previous analyzes of uncertainty and worth.

The findings from this study and its conceptual contribution emphasize the need for a more detailed, as well as a more comprehensive, understanding of how scholars cope with uncertainties about their future. Ways of coping with uncertainties privilege certain ways of reasoning and preference to act but also certain identity positions and ways of developing a sense of self. As we try to make sense of how intensified measurements of performance and increasingly precarious working conditions are impacting the lives of academics, we should thus not forget that “scholarship is far from being an abstract or disconnected pursuit; instead, it is intimately tied to the image that academics hold of themselves […] and how they think they should lead their lives” (Lamont 2009: 195). Trying to capture this dynamic, the analysis of how appraisal devices inform the research and career practices of scholars opens up an analytical opportunity to understand the intricate ways in which valuing, being, and knowing are entangled in academic life.
